# Data Augmentation for Brain-Tumor Segmentation: A Review

**DOI:** 10.3389/fncom.2019.00083

**Published:** 2019-12-11

**Authors:** Jakub Nalepa, Michal Marcinkiewicz, Michal Kawulok

**Affiliations:** ^1^Future Processing, Gliwice, Poland; ^2^Silesian University of Technology, Gliwice, Poland; ^3^Netguru, Poznan, Poland

**Keywords:** MRI, image segmentation, data augmentation, deep learning, deep neural network

## Abstract

Data augmentation is a popular technique which helps improve generalization capabilities of deep neural networks, and can be perceived as implicit regularization. It plays a pivotal role in scenarios in which the amount of high-quality ground-truth data is limited, and acquiring new examples is costly and time-consuming. This is a very common problem in medical image analysis, especially tumor delineation. In this paper, we review the current advances in data-augmentation techniques applied to magnetic resonance images of brain tumors. To better understand the practical aspects of such algorithms, we investigate the papers submitted to the Multimodal Brain Tumor Segmentation Challenge (BraTS 2018 edition), as the BraTS dataset became a standard benchmark for validating existent and emerging brain-tumor detection and segmentation techniques. We verify which data augmentation approaches were exploited and what was their impact on the abilities of underlying supervised learners. Finally, we highlight the most promising research directions to follow in order to synthesize high-quality artificial brain-tumor examples which can boost the generalization abilities of deep models.

## 1. Introduction

Deep learning has established the state of the art in many sub-areas of computer vision and pattern recognition (Krizhevsky et al., 2017), including medical imaging and medical image analysis (Litjens et al., 2017). Such techniques automatically discover the underlying data representation to build high-quality models. Although it is possible to utilize generic priors and exploit domain-specific knowledge to help improve representations, deep features can capture very discriminative characteristics and explanatory factors of the data which could have been omitted and/or unknown for human practitioners during the process of manual feature engineering (Bengio et al., 2013).

In order to successfully build well-generalizing deep models, we need huge amount of ground-truth data to avoid overfitting of such large-capacity learners, and “memorizing” training sets (LeCun et al., 2016). It has become a significant obstacle which makes deep neural networks quite challenging to apply in the medical image analysis field where acquiring *high-quality* ground-truth data is time-consuming, expensive, and very human-dependent, especially in the context of brain-tumor delineation from magnetic resonance imaging (MRI) (Isin et al., 2016; Angulakshmi and Lakshmi Priya, 2017; Marcinkiewicz et al., 2018; Zhao et al., 2019). Additionally, the majority of manually-annotated image sets are imbalanced—examples belonging to some specific classes are often under-represented. To combat the problem of limited medical training sets, data augmentation techniques, which generate synthetic training examples, are being actively developed in the literature (Hussain et al., 2017; Gibson et al., 2018; Park et al., 2019).

In this review paper, we analyze the brain-tumor segmentation approaches available in the literature, and thoroughly investigate which techniques have been utilized by the participants of the Multimodal Brain Tumor Segmentation Challenge (BraTS 2018). To the best of our knowledge, the dataset used for the BraTS challenge is currently the largest and the most comprehensive brain-tumor dataset utilized for validating existent and emerging algorithms for detecting and segmenting brain tumors. Also, it is heterogeneous in the sense that it includes both low- and high-grade lesions, and the included MRI scans have been acquired at different institutions (using different MR scanners). We discuss the brain-tumor data augmentation techniques already available in the literature, and divide them into several groups depending on their underlying concepts (section 2). Such MRI data augmentation approaches have been applied to augment other datasets as well, also acquired for different organs (Amit et al., 2017; Nguyen et al., 2019; Oksuz et al., 2019).

In the BraTS challenge, the participants are given multi-modal MRI data of brain-tumor patients (as already mentioned, both low- and high-grade gliomas), alongside the corresponding ground-truth multi-class segmentation (section 3). In this dataset, different sequences are co-registered to the same anatomical template and interpolated to the same resolution of 1 mm^3^. The task is to build a supervised learner which is able to generalize well over the unseen data which is released during the testing phase. In section 4, we summarize the augmentation methods reported in 20 papers published in the BraTS 2018 proceedings. Here, we focused on those papers which *explicitly* mentioned that the data augmentation had been utilized, and clearly stated what kind of data augmentation had been applied. Although such augmentations are single-modal—meaning that they operate over the MRI from a single sequence—they can be easily applied to co-registered series, hence to augment multi-modal tumor examples. Finally, the paper is concluded in section 5, where we summarize the advantages and disadvantages of the reviewed augmentation techniques, and highlight the promising research directions which emerge from (not only) BraTS.

## 2. Data Augmentation for Brain-Tumor Segmentation

**Figure 1:**
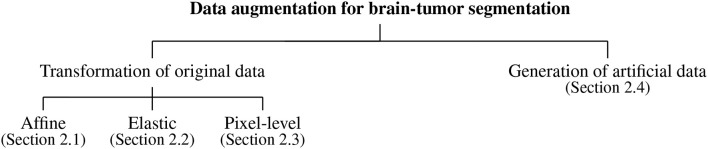
Data augmentation for brain-tumor segmentation—a taxonomy.

Data augmentation algorithms for brain-tumor segmentation from MRI can be divided into the following main categories (which we render in a taxonomy presented in Figure 1): the algorithms exploiting various transformations of the original data, including *affine* image transformations (section 2.1), *elastic* transformations (section 2.2), *pixel-level* transformations (section 2.3), and various approaches for *generating artificial data* (section 2.4). In the following subsections, we review the approaches belonging to all groups of such augmentation methods in more detail.

**Figure 2:**
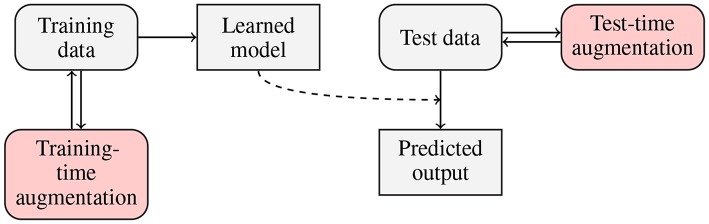
Flowchart presenting training- and test-time data augmentation. In the training-time data augmentation approach, we generate synthetic data to increase the representativeness of a training set (and ultimately build better models), whereas in test-time augmentation, we benefit from the ensemble-like technique, in which multiple homogeneous classifiers vote for the final class label for an incoming example by classifying this sample and a number of its augmented versions.

Traditionally, data augmentation approaches have been applied to increase the size of training sets, in order to allow large-capacity learners benefit from more representative training data (Wong et al., 2016). There is, however, a new trend in the deep learning literature, in which examples are augmented on the fly (i.e., during the inference), in the *test-time*^1^ augmentation process. In Figure 2, we present a flowchart in which both training- and test-time data augmentation is shown. Test-time data augmentation can help increase the *robustness* of a trained model by simulating the creation of a homogeneous ensemble, where (*n*+1) models (of the same type, and trained over the same training data) *vote* for the final class label of an incoming test example, and *n* denotes the number of artificially-generated samples, elaborated for the test example which is being classified. The robustness of a deep model is often defined as its ability to correctly classify previously unseen examples—such incoming examples are commonly “noisy” or slightly “perturbed” when confronted with the original data, therefore they are more challenging to classify and/or segment (Rozsa et al., 2016). Test-time data augmentation can be exploited for estimating the level of uncertainty of deep networks during the inference—it brings new exciting possibilities in the context of medical image analysis, where quantifying the robustness and deep-network reliability are crucial practical issues (Wang et al., 2019). This type of data augmentation can utilize those methods which *modify* an incoming example, e.g., by applying affine, pixel-level or elastic transformations in the case of brain-tumor segmentation from MRI.

### 2.1. Data Augmentation Using Affine Image Transformations

**Figure 3:**
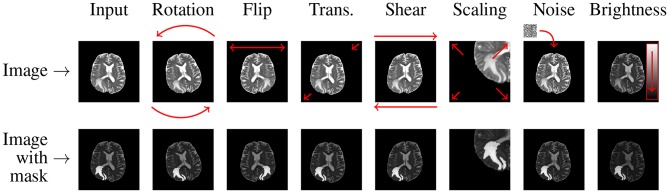
Applying affine and pixel-level (discussed in more detail in section 2.3) transformations can help significantly increase the size (and potentially representativeness) of training sets. In this example, we generate seven new images based on the original MRI (coupled with its ground truth in the bottom row).

In the *affine approaches*, existent image data undergo different operations (rotation, zooming, cropping, flipping, or translations) to increase the number of training examples (Pereira et al., 2016; Liu et al., 2017). Shin et al. pointed out that such traditional data augmentation techniques fundamentally produce very correlated images (Shin et al., 2018), therefore can offer very little improvements for the deep-network training process and future generalization over the unseen test data (such examples do not regularize the problem sufficiently). Additionally, they can also generate anatomically incorrect examples, e.g., using rotation. Nevertheless, affine image transformations are trivial to implement (in both 2D and 3D), they are fairly flexible (due to their hyper-parameters), and are widely applied in the literature. In an example presented in Figure 3, we can see that applying simple data augmentation techniques can lead to a significant increase in the number of training samples.

#### 2.1.1. Flip and Rotation

Random flipping creates a mirror reflection of an original image along one (or more) selected axis. Usually, natural images can be flipped along the horizontal axis, which is not the case for the vertical one because up and down parts of an image are not always “interchangeable.” A similar property holds for MRI brain images—in the axial plane a brain has two hemispheres, and the brain (in most cases) can be considered anatomically symmetrical. Flipping along the horizontal axis swaps the left hemisphere with the right one, and vice versa. This operation can help various deep classifiers, especially those benefitting from the contextual tumor information, be invariant with respect to their position within the brain which would be otherwise difficult for not representative training sets (e.g., containing brain tumors located only in the left or right hemisphere). Similarly, rotating an image by an angle α around the center pixel can be exploited in this context. This operation is followed by appropriate interpolation to fit the original image size. The rotation operation denoted as *R* is often coupled with zero-padding applied to the missing pixels:

(1) $$\begin{array}{r} R=\left(\begin{array}{cc} \cos\alpha & -\sin\alpha \\ \sin\alpha & \cos\alpha \end{array}\right). \end{array}$$

#### 2.1.2. Translation

The translation operation shifts the entire image by a given number of pixels in a chosen direction, while applying padding accordingly. It allows the network to not become focused on features present mainly in one particular spatial region, but it forces the model to learn spatially-invariant features instead. As in the case of rotation—since the MRI scans of different patients available in training sets are often not co-registered—translation of an image by a given number of pixels along a selected axis (or axes) can create useful and viable images. However, this procedure may not be “useful” for all deep architectures—convolutional neural networks exploit convolutions and pooling operations, which are intrinsically spatially-invariant (Asif et al., 2018).

#### 2.1.3. Scaling and Cropping

Introducing scaled versions of the original images into the training set can help the deep network learn valuable deep features independently of their original scale. This operation *S* can be performed independently in different directions (for brevity, we have only two dimensions here):

(2) $$\begin{array}{r} S=\left(\begin{array}{cc} s_{x} & 0\\ 0 & s_{y} \end{array}\right), \end{array}$$

and the scaling factors are given as *s*_*x*_ and *s*_*y*_ for the *x* and *y* directions, respectively. As tumors vary in size, scaling can indeed bring viable augmented images into a training set. Since various deep architectures require images of the constant size, scaling is commonly paired with cropping to maintain the original image dimensions. Such augmented brain-tumor examples may manifest tumoral features at different scales. Also, cropping can limit the field of view only to those parts of the image which are important (Menze et al., 2015).

#### 2.1.4. Shearing

The shear transformation (*H*) displaces each point in an image in a selected direction. This displacement is proportional to its distance from the line which goes through the origin and is parallel to this direction:

(3) $$\begin{array}{r} H=\left(\begin{array}{cc} 1 & h_{x}\\ h_{y} & 1 \end{array}\right), \end{array}$$

where *h*_*x*_ and *h*_*y*_ denote the shear coefficient in the *x* and *y* directions, respectively (as previously, we consider two dimensions for readability). Although this operation can deform shapes, it is rarely used to augment medical image data because we often want to preserve original shape characteristics (Frid-Adar et al., 2018).

### 2.2. Data Augmentation Using Elastic Image Transformations

Data augmentation algorithms based on unconstrained *elastic transformations* of training examples can introduce shape variations. They can bring lots of noise and damage into the training set if the deformation field is seriously varied—see an example by Mok and Chung (2018) in which a widely-used elastic transform produced a totally unrealistic synthetic MRI scan of a human brain. If the simulated tumors were placed in “unrealistic” positions, it would likely force the segmentation engine to become invariant to contextual information and rather focus on the lesion's appearance features (Dvornik et al., 2018). Although there are works which indicate that such aggressive augmentation may deteriorate the performance of the models in brain-tumor delineation (Lorenzo et al., 2019), it is still an open issue. Chaitanya et al. (2019) showed that visually non-realistic synthetic examples can improve the segmentation of cardiac MRI and noted that it is slightly counter-intuitive—it may have occurred due to the inherent structural and deformation-related characteristics of the cardiovascular system. Finally, elastic transformations often benefit from B-splines (Huang and Cohen, 1996; Gu et al., 2014) or random deformations (Castro et al., 2018).

Diffeomophic mappings play an important role in brain imaging, as they are able to preserve topology and generate biologically plausible deformations. In such transformations, the *diffeomorphism ϕ* (also referred to as a *diffeomorphic mapping*) is given in the spatial domain Ω of a source image *I*, and transforms *I* to the target image *J*: *I* ◦ *ϕ*^−1^(**x**, 1). The mapping is the solution of the differential equation:

(4) $$\begin{array}{r} \frac{d\phi \left(x,t\right)}{dt}=v\left(\phi \left(x,t\right),t\right), \end{array}$$

where *ϕ*(***x***, 0) = **x**, *v* is a time-dependent smooth velocity field, $v:\Omega \times t\to R^{d}$, *ϕ*(**x**, *t*) is a geodesic path (*d* denotes the dimensionality of the spatial domain Ω), and *ϕ*(*x, t*):Ω × *t* → Ω. In Nalepa et al. (2019a), we exploited the directly manipulated free-form deformation, in which the velocity vector fields are regularized using B-splines (Tustison et al., 2009). The *d*-dimensional update field δ*v*_*i*_1_, …, *i*_*d*__ is

(5) $$\begin{array}{r} \delta v_{i_{1},\ldots ,i_{d}}=\frac{\overset{N_{\Omega }}{\underset{c=1}{\sum }}{\left(\frac{\partial \xi }{\partial x}\right)}_{c}\overset{d}{\underset{j=1}{\prod }}B_{i_{j}}\left(x_{j}^{c}\right)\overset{d}{\underset{j=1}{\prod }}B_{i_{j}}^{2}\left(x_{j}^{c}\right)}{\left(\overset{N_{\Omega }}{\underset{c=1}{\sum }}\overset{d}{\underset{j=1}{\prod }}B_{i_{j}}^{2}\left(x_{j}^{c}\right)\right)\left(\overset{r+1}{\underset{k_{1}=1}{\sum }}\ldots \overset{r+1}{\underset{k_{d}=1}{\sum }}\overset{d}{\underset{j=1}{\prod }}B_{k_{j}}^{2}\left(x_{j}^{c}\right)\right)}, \end{array}$$

and *B*(·) are the B-spline basis functions, *N*_Ω_ denotes the number of pixels in the domain of the reference image, *r* is the spline order (in all dimensions), and $\frac{\partial \xi }{\partial x}$ is the gradient of the spatial similarity metric at a pixel *c*. The B-spline functions act as regularizers of the solution for each parametric dimension (Tustison and Avants, 2013).

**Figure 4:**
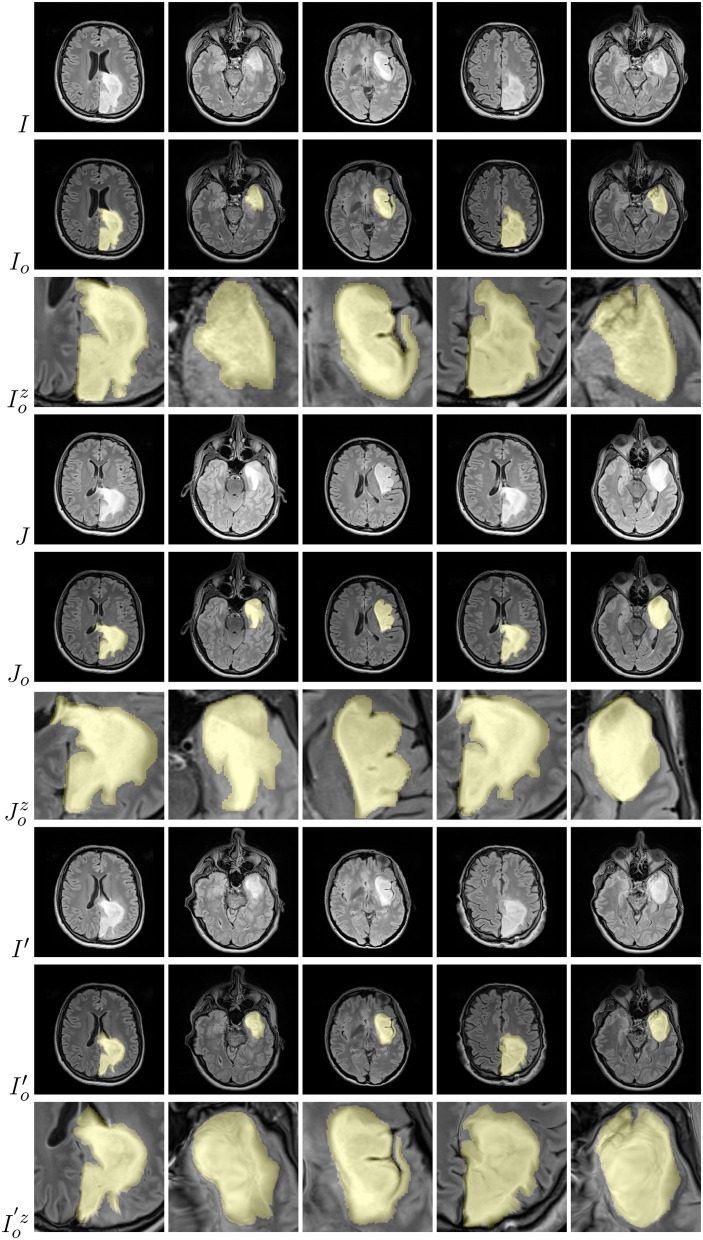
Diffeomorphic image registration applied to example brain images allowed for obtaining visually-plausible generated images. For source (*I*), target (*J*), and artificially generated (*I*′) images, we also present tumor masks overlayed over the corresponding original images (in yellow; rows with the *o* subscript), alongside a zoomed part of a tumor (rows with the *z* superscript).

**Figure 5:**
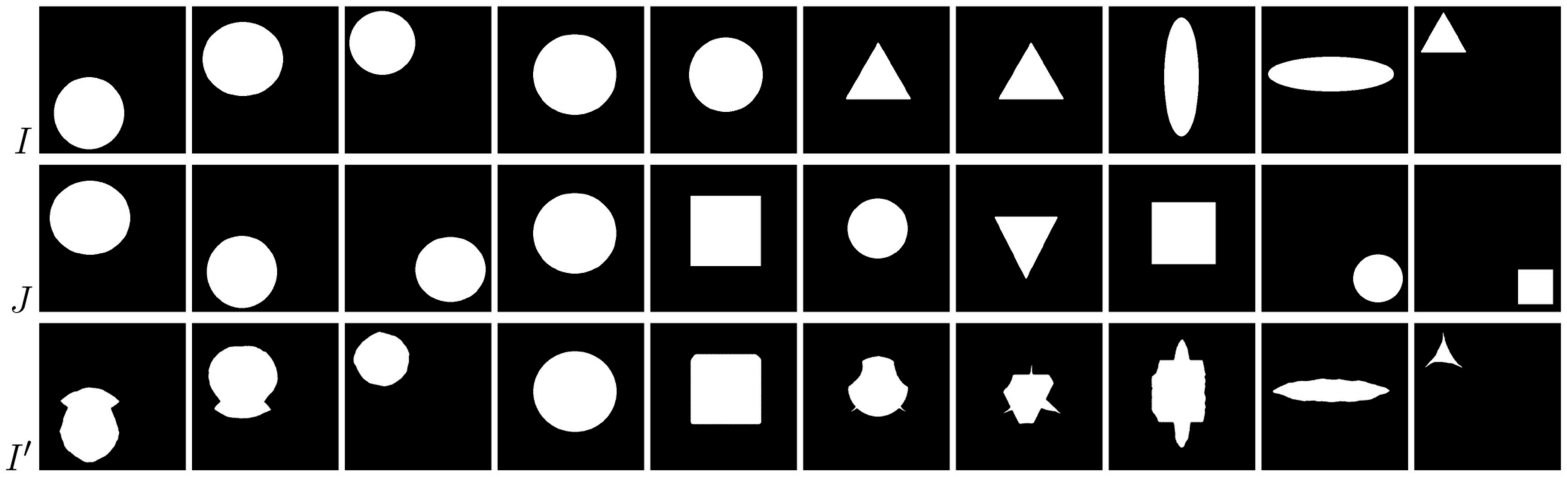
Diffeomorphic image registration applied to basic shapes which underwent simple affine registration (translation) before diffeomorphic mapping. Source images (*I*) transformed to match the corresponding targets (*J*) still clearly expose their spatial characteristics (*I*′).

Examples of brain-tumor images generated using diffeomorphic registration are given in Figure 4—such artificially-generated data significantly improved the abilities of deep learners, especially when combined with affine transformations, as we showed in Nalepa et al. (2019a). The generated (*I*′) images preserve topological information of the original image data (*I*) with subtle changes to the tissue. Diffeomorphic registration may be applied not only to images exposing anatomical structures (Tward and Miller, 2017). In Figure 5, we present examples of simple shapes which underwent this transformation—the topological information is clearly maintained in the generated images as well.

### 2.3. Data Augmentation Using Pixel-Level Image Transformations

There exist augmentation techniques which do not alter geometrical shape of an image (therefore, all geometrical features remain unchanged during the augmentation process), but affect the pixel intensity values (either locally, or across the entire image). Such operations can be especially useful in medical image analysis, where different training images are acquired in different locations and using different scanners, hence can be intrinsically heterogeneous in the pixel intensities, intensity gradients or “saturation”^2^. During the *pixel-level augmentation*, the pixel intensities are commonly perturbed using either *random* or zero-mean *Gaussian* noise (with the standard deviation corresponding to the appropriate data dimension), with a given probability (the former operation is referred to as the *random intensity variation*). Other pixel-level operations include shifting and scaling of pixel-intensity values (and modifying the image brightness), applying gamma correction and its multiple variants (Agarwal and Mahajan, 2017; Sahnoun et al., 2018), sharpening, blurring, and more (Galdran et al., 2017). This kind of data augmentation is often exploited for high-dimensional data, as it can be conveniently applied to selected dimensions (Nalepa et al., 2019b).

### 2.4. Data Augmentation by Generating Artificial Data

**Figure 6:**
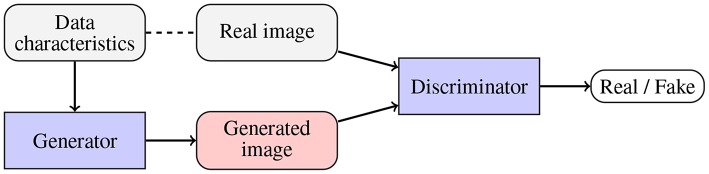
Generative adversarial networks are aimed at generating fake data (by a *generator*; potentially using some available data characteristics) which is indistinguishable from the original data by the *discriminator*. Therefore, the generator and discriminator compete with one another.

To alleviate the problems related to the basic data augmentation approaches (including the problem of generating correlated data samples), various approaches toward *generating artificial data* (GAD) have been proposed. Generative adversarial networks (GANs), originally introduced in Goodfellow et al. (2014), are being exploited to augment medical datasets (Han et al., 2019; Shorten and Khoshgoftaar, 2019). The main objective of a GAN (Figure 6) is to generate a new data example (by a *generator*) which will be indistinguishable from the real data by the *discriminator* (the generator competes with the discriminator, and the overall optimization mimics the min-max game). Mok and Chung proposed a new GAN architecture which utilizes a coarse-to-fine generator whose aim is to capture the manifold of the training data and generate augmented examples (Mok and Chung, 2018). Adversarial networks have been also used for semantic segmentation of brain tumors (Rezaei et al., 2017), brain-tumor detection (Varghese et al., 2017), and image synthesis of different modalities (Yu et al., 2018). Although GANs allow us to introduce invariance and robustness of deep models with respect to not only affine transforms (e.g., rotation, scaling, or flipping) but also to some shape and appearance variations, convergence of the adversarial training and existence of its equilibrium point remain the open issues. Finally, there exist scenarios in which the generator renders multiple very similar examples which cannot improve the generalization of the system—it is known as the *mode collapse problem* (Wang et al., 2017).

An interesting approach for generating phantom image data was exploited in Gholami et al. (2018), where the authors utilized a multi-species partial differential equations (PDE) growth model of a tumor to generate synthetic lesions. However, such data does not necessarily follow the correct intensity distribution of a real MRI, hence it should be treated as a separate modality, because using the artificial data which is sampled from a very different distribution may adversely affect the overall segmentation performance by “tricking” the underlying deep model (Wei et al., 2018). The tumoral growth model itself captured the time evolution of enhancing and necrotic tumor concentrations together with the edema induced by a tumor. Additionally, the deformation of a lesion was simulated by incorporating the linear elasticity equations into the model. To deal with the different data distributions, the authors applied CycleGAN (Zhu et al., 2017) for performing domain adaptation (from the generated phantom data to the real BraTS MRI scans). The experimental results showed that the domain adaptation was able to generate images which were practically indistinguishable from the real data, therefore could be safely included in the training set.

A promising approach of combining training samples using their linear combinations (referred to as *mixup*) was proposed by Zhang et al. (2017), and further enhanced for medical image segmentation by Eaton-Rosen et al. in their *mixmatch* algorithm (Eaton-Rosen et al., 2019), which additionally introduced a technique of selecting training samples that undergo linear combination. Since the medical image datasets are often imbalanced (with the tumorous examples constituting the minority class), training patches with highest “foreground amounts” (i.e., the number of pixels annotated as tumorous) are combined with those with the lowest concentration of foreground. The authors showed that their approach can increase performance in medical-image segmentation tasks, and related its success to the mini-batch training. It is especially relevant in the medical-image analysis, because the sizes of input scans are usually large, hence the batches are small to keep the training memory requirements feasible in practice. Such data-driven augmentation techniques can also benefit from growing ground-truth datasets (e.g., BraTS) which manifest large variability of brain tumors, to generate even more synthetic examples. Also, they could be potentially applied at test time to build an ensemble-like model, if a training patch/image which matches the test image being classified was efficiently selected from the training set.

## 3. Data

In this work, we analyzed the approaches which were exploited by the BraTS 2018 participants to segment brain tumors from MRI (45 methods have been published, Crimi et al., 2019), and verified which augmentation scenarios were exploited in these algorithms. All of those techniques have been trained over the BraTS 2018 dataset consisting of MRI-DCE data of 285 patients with diagnosed gliomas: 210 patients with high-grade glioblastomas (HGG), and 75 patients with low-grade gliomas (LGG), and validated using the validation set of 66 previously unseen patients (both LGG and HGG, however the grade has not been revealed) (Menze et al., 2015; Bakas et al., 2017a,b,c). Each study was manually annotated by one to four expert readers. The data comes in four co-registered modalities: native pre-contrast (T1), post-contrast T1-weighted (T1c), T2-weighted (T2), and T2 Fluid Attenuated Inversion Recovery (FLAIR). All the pixels have one of four labels attached: healthy tissue, Gd-enhancing tumor (ET), peritumoral edema (ED), the necrotic and non-enhancing tumor core (NCR/NET). The scans were skull-stripped and interpolated to the same shape (155, 240, 240 with the voxel size of 1 mm^3^).

Importantly, this dataset manifests very heterogeneous image quality, as the studies were acquired across different institutions, and using different scanners. On the other hand, the delineation procedure was clearly defined which allowed for obtaining similar ground-truth annotations across various readers. To this end, the BraTS dataset—as the largest, most heterogeneous, and carefully annotated set—has been established as a standard brain-tumor dataset for quantifying the performance of existent and emerging detection and segmentation approaches. This heterogeneity is pivotal, as it captures a wide range of tumor characteristics, and the models trained over BraTS are easily applicable for segmenting other MRI scans (Nalepa et al., 2019).

To show this desirable feature of the BraTS set experimentally, we trained our U-Net-based ensemble architecture (Marcinkiewicz et al., 2018) using (a) BraTS 2019 training set (exclusively FLAIR sequences) and (b) our set of 41 LGG (WHO II) brain-tumor patients who underwent the MR imaging with a MAGNETOM Prisma 3T system (Siemens, Erlangen, Germany) equipped with a maximum field gradient strength of 80 mT/m, and using a 20-channel quadrature head coil. The MRI sequences were acquired in the axial plane with a field of view of 230 × 190 mm, matrix size 256 × 256 and 1 mm slice thickness with no slice gap. In particular, we exploited exclusively FLAIR series with TE = 386 ms, TR = 5,000 ms, and inversion time of 1,800 ms for segmentation of brain tumors. These scans underwent the same pre-processing as applied in the case of BraTS, however they were *not* segmented following the same delineation protocol, hence the characteristics of the manual segmentation likely differ across (a) and (b). The 4-fold cross-validation showed that although the deep models trained over (a) and (b) gave the statistically different results at *p* < 0.001, according to the two-tailed Wilcoxon test^3^, the ensemble of models trained over (a) correctly detected 71.4% (5/7 cases) of brain tumors in the WHO II test dataset, which included seven patients kept aside while building an ensemble, with the average whole-tumor DICE of 0.80, where DICE is given as:

(6) $$\begin{array}{r} \text{DICE}\left(\text{A},\text{ B}\right)=\frac{2\cdot |A\cap B|}{\left|A|+|B\right|}, \end{array}$$

where *A* and *B* are two segmentations, i.e., manual and automated, 0 ≤ DICE ≤ 1, and DICE = 1 means the perfect segmentation score. On the other hand, a deep model trained over the WHO II training set and used for segmenting the test WHO II cases detected 85.7% tumors (6/7 patients) with the average whole-tumor DICE = 0.84. This tiny experiment shows that the segmentation engines trained over BraTS can capture tumor characteristics which are manifested in MRI data acquired and analyzed using different protocols, and allow us to obtain high-quality segmentation. Interestingly, if we train our ensemble over the combined BraTS 2019 and WHO II training sets, we will end up having the correct detection of 85.7% tumors (6/7 cases) with the average whole-tumor DICE of 0.76. We can appreciate the fact that we were able to improve the detection, but the segmentation quality slightly dropped, showing that the detected case was challenging to segment. Finally, it is worth mentioning that this experiment sheds only some light on the effectiveness of applying the deep models (or other data-driven techniques) trained over BraTS for analyzing different MRI brain images. The manual delineation protocols were different, and the lack of inter-rater agreement may play pivotal role in quantifying automated segmentation algorithms over such differently acquired and analyzed image sets—it is unclear if the differences result from the inter-rater disagreement of the incorrect segmentation (Hollingworth et al., 2006; Fyllingen et al., 2016; Visser et al., 2019).

### 3.1. Example BraTS Images

**Figure 7:**
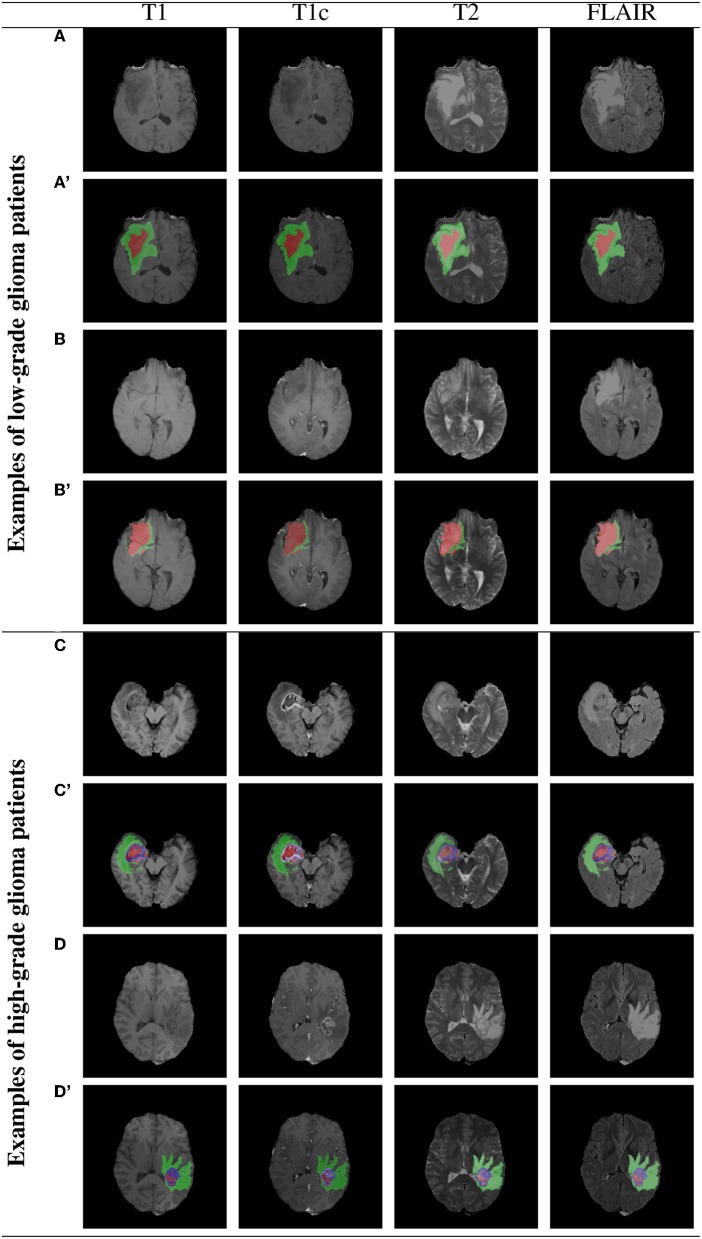
Two example low- and high-grade glioma patients from the BraTS 2018 dataset: red—GD-enhancing tumor (ET), green—peritumoral edema (ED), and blue—necrotic and non-enhancing tumor core (NCR/NET); **(A–D)** show original images, whereas **(A'–D')** present overlaid ground-truth masks.

Example BraTS 2018 images are rendered in Figure 7 (two low-grade and two high-grade glioma patients), alongside the corresponding multi-class ground-truth annotations. We can appreciate that different parts of the tumors are manifested in different modalities—e.g., necrotic and non-enhancing tumor core is typically hypo-intense in T1-Gd when compared to T1 (Bakas et al., 2018). Therefore, multi-modal analysis appears crucial to fully benefit from the available image information.

## 4. Brain-Tumor Data Augmentation in Practice

### 4.1. BraTS 2018 Challenge

The BraTS challenge is aimed at evaluating the state-of-the-art approaches toward accurate multi-class brain-tumor segmentation from MRI. In this work, we review all *published* methods which were evaluated within the framework of the BraTS 2018 challenge—although 61 teams participated in the testing phase (Bakas et al., 2018), only 45 methods were finally described and published in the post-conference proceedings (Crimi et al., 2019). We verify which augmentation techniques were exploited to help boost generalization abilities of the proposed supervised learners. We exclusively focus on 20 papers (44% of all manuscripts) in which the authors *explicitly* stated that the augmentation had been used and report the type of the applied augmentation.

In Table 1, we summarize all investigated brain-tumor segmentation algorithms, and report the deep models utilized in the corresponding works alongside the augmentation techniques. In most of the cases, the authors followed the cross-validation scenario, and divided the training set into multiple non-overlapping folds. Then, separate models were trained over such folds, and the authors finally formed an ensemble of heterogeneous classifiers (trained over different training data) to segment previously unseen test brain-tumor images. Also, there are approaches, e.g., by Albiol et al. (2019), Chandra et al. (2018), or Sun et al. (2018), in which a variety of deep neural architectures were used.

**Table 1:** Data augmentation techniques applied in the approaches validated within the BraTS 2018 challenge framework.

**References**	**Model**	**Flip**	**Rot**.	**Trans**.	**Scale**	**Shear**	**Elastic**	**GAD**	**Pixel-wise**
Albiol et al., 2019	VGG, Inception, Dense	3D affine transformations							
Benson et al., 2018^*^	CNN (encoder-decoder)	Yes							Random
Carver et al., 2018	U-Net	Yes							
Chandra et al., 2018	V-Net, ResNet-18, FC-CRF	Yes			Yes				
Dai et al., 2018	Domain-adapted U-Net	Yes							
Feng et al., 2018	U-Net	Yes							
Gholami et al., 2018^*^	U-Net							PDE	
Isensee et al., 2018	U-Net	Yes	Yes		Yes		Random		Gamma
Kao et al., 2018	DeepMedic, 3D U-Net	Yes							
Kermi et al., 2018	U-Net	Yes	Yes	Yes					
Lachinov et al., 2018^*^	Cascaded U-Net	Yes					B-spline		Gaussian
Ma and Yang, 2018	3D CNN	Yes	Yes		Yes				
McKinley et al., 2018	Dense CNN	Yes	Yes						Shift, scale
Mehta and Arbel, 2018	U-Net		Yes	Yes	Yes	Yes			
Myronenko, 2018	CNN (encoder-decoder)	Yes			Yes				Shift
Nuechterlein and Mehta, 2018	3D-ESPNet	Yes			Yes				
Puybareau et al., 2018	VGG-16		Yes		Yes				
Rezaei et al., 2018^†^	Voxel-GAN		Yes		Yes				Gaussian
Sun et al., 2018	CNN, DFKZ, 3D CNN	Yes							Gaussian
Wang et al., 2018^*^^†^	CNN	Yes	Yes		Yes				Random
Number of methods utilizing this augmentation →		15	8	2	9	1	2	1	8
Percentage (%) of methods utilizing this augmentation →		75	40	10	45	5	10	5	40

*The top-performing techniques (over the unseen test set) are annotated with green*.

* *The authors verified the impact of data augmentation of the generalization abilities of their deep models*.

† *The authors used both training- and test-time data augmentation*.

In the majority of investigated brain-tumor segmentation techniques, the authors applied relatively simple training-time data augmentation strategies—the combination of training- and test-time augmentation was used only in two methods (Rezaei et al., 2018; Wang et al., 2018). In 75% of the analyzed approaches, random flipping was executed to increase the training set size and provide anatomically correct brain images^4^. Similarly, rotating and scaling MRI images was applied in 40% and 45% of techniques, respectively. Since modern deep network architectures are commonly translation-invariant, this type of affine augmentation was used only in two works. Although other augmentation strategies were not as popular as easy-to-implement affine transformations, it is worth noting that the pixel-wise operations were utilized in all of the top-performing techniques (the algorithms by Myronenko (2018), Isensee et al. (2018), and McKinley et al. (2018) achieved the first, second, and third place across all segmentation algorithms^5^, respectively). Additionally, Isensee et al. (2018) exploited elastic transformations in their aggressive data augmentation procedure which significantly increased the size and representativeness of their training sets, and ultimately allowed for outperforming a number of other learners. Interestingly, the authors showed that the state-of-the-art U-Net architecture can be extremely competitive with other (much deeper and complex) models if the data is appropriately curated. It, in turn, manifests the importance of data representativeness and quality in the context of robust medical image analysis.

**Figure 8:**
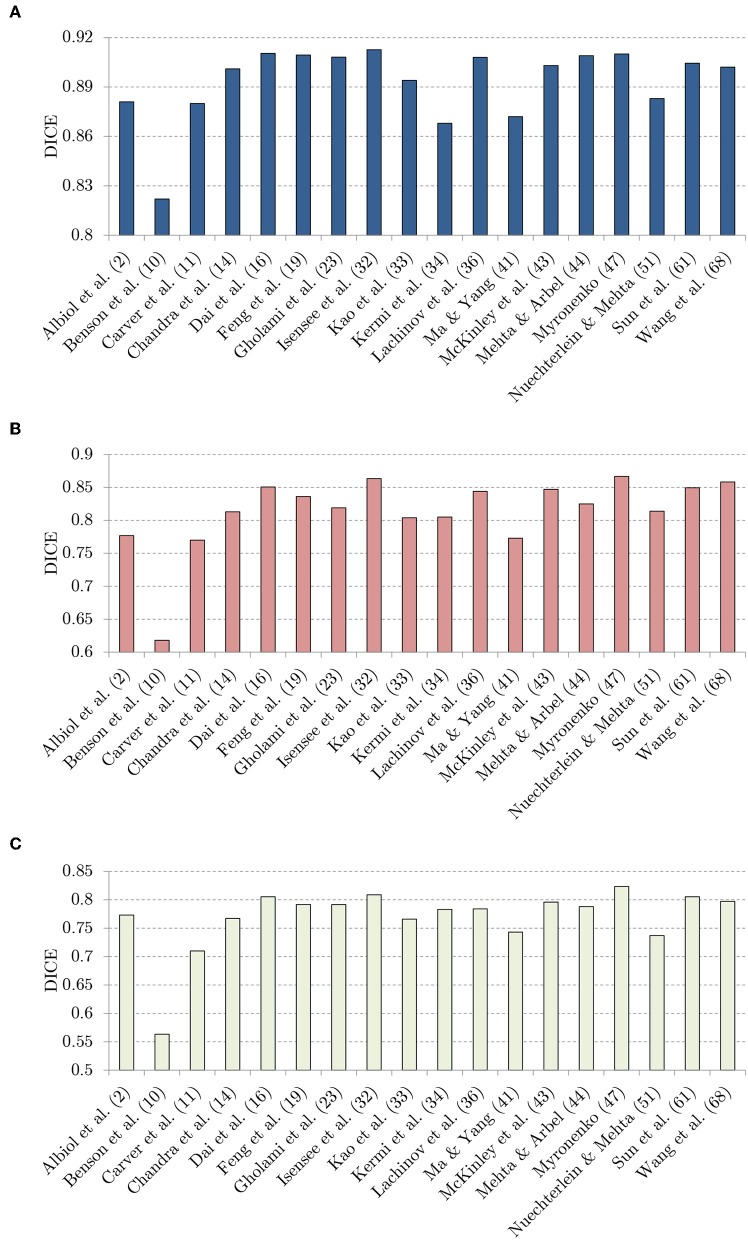
The DICE values: **(A)** whote-tumor (WT), **(B)** tumor core (TC), and **(C)** enhancing tumor (ET), obtained using the investigated techniques over the BraTS 2018 validation set.

In Figure 8, we visualize the DICE scores obtained using almost all investigated methods (Puybareau et al., 2018; Rezaei et al., 2018 did not report the results over the unseen BraTS 2018 validation set, therefore these methods are not included in the figure). It is worth mentioning that the trend is fairly coherent for all classes (whole tumor, tumor core, and enhancing tumor), and the best-performing methods by Isensee et al. (2018), McKinley et al. (2018), and Myronenko (2018) consistently outperform the other techniques in all cases. Although the success of these approaches obviously lies not only in the applied augmentation techniques, it is notable that the authors extensively benefit from generating additional synthetic data.

Albeit data augmentation is introduced in order to improve the generalization capabilities of supervised learners, this impact was verified only in four BraTS 2018 papers (Benson et al., 2018; Gholami et al., 2018; Lachinov et al., 2018; Wang et al., 2018). Gholami et al. (2018) showed that their PDE-based augmentation delivers very significant improvement in the DICE scores obtained for segmenting all parts of the tumors in the multi-class classification. The same performance boost (in the DICE values obtained for each class) was reported by Lachinov et al. (2018). Finally, Wang et al. (2018) showed that the proposed test-time data augmentation led to improving the performance of their convolutional neural networks.

In Table 2, we gathered the DICE scores obtained with and without the corresponding data augmentation, alongside the change in DICE (reported in %; the larger the DICE score becomes, the better segmentation has been obtained). Interestingly, training-time data augmentation appeared to be adversely affecting the performance of the algorithm presented by Benson et al. (2018). On the other hand, the authors showed that the Hausdorff distance, being the maximum distance of all points from the segmented lesion to the corresponding nearest point of the ground-truth segmentation (Sauwen et al., 2017), significantly dropped, hence the maximum segmentation error quantified by this metric was notably reduced (the smaller the Hausdorff distance becomes, the better segmentation has been elaborated; Table 3). Test-time data augmentation exploited by Wang et al. (2018) not only decreased DICE for the whole-tumor segmentation, but also caused the increase of the correspoding Hausdorff distance. Therefore, applying it in the WT segmentation scenario led to decreasing the abilities of the underlying models. Overall, the vast majority of methods neither report nor analyze the real impact of the incorporated augmentation techniques on the classification performance and/or inference time of their deep models. Although we believe the authors did investigate the advantages (and disadvantages) of their data generation strategies (either experimentally or theoretically), data augmentation is often used a standard tool which is applied to any *difficult* data (e.g., imbalanced, with highly under-represented classes).

**Table 2:** The impact of applying data augmentation on the average DICE scores.

	**Without augmentation**			**With augmentation**			**Change (in %**)		
**References**	**WT**	**TC**	**ET**	**WT**	**TC**	**ET**	**ΔWT**	**ΔTC**	**ΔET**
Benson et al., 2018	0.82	0.64	0.59	0.82	0.61	0.56	0	−5	−5
Gholami et al., 2018	0.89	0.80	0.74	0.91	0.82	0.79	+2	+3	+7
Lachinov et al., 2018^*^	0.91	0.84	0.77	0.91	0.84	0.78	0	0	+1
Wang et al., 2018	0.90	0.85	0.79	0.90	0.86	0.80	0	+1	+1

*For the methods reported by Lachinov et al. (2018) and Wang et al. (2018), we analyzed the best-performing models*.

* *The authors verified the impact of data augmentation over the training set*.

**Table 3:** The impact of applying data augmentation on the average Hausdorff distance values (in mm).

	**Without augmentation**			**With augmentation**			**Change (in %)**		
**References**	**WT**	**TC**	**ET**	**WT**	**TC**	**ET**	**ΔWT**	**ΔTC**	**ΔET**
Benson et al., 2018	94.28	130.70	18.12	13.57	17.95	14.29	−86	−86	−21
Wang et al., 2018	5.38	6.61	3.34	6.18	6.37	3.13	+26	−4	−6

*For the method reported by Wang et al. (2018), we analyzed the best-performing models. Note that Gholami et al. (2018) and Lachinov et al. (2018) did not present the Hausdorff distances obtained using their approaches*.

### 4.2. Beyond the BraTS Challenge

Although practically all brain-tumor segmentation algorithms which emerge in the recent literature have been tested over the BraTS datasets, we equipped our U-Nets with a battery of augmentation techniques (summarized in Table 4) and verified their impact over our clinical MRI data in Lorenzo et al. (2019). In this experiment, we have focused on the whole-tumor segmentation, as it was an intermediate step in the automated dynamic contrast-enhanced MRI analysis, in which perfusion parameters have been extracted for the entire tumor volume. Additionally, this dataset was manually delineated by a reader (8 years of experience) who highlighted the whole-tumor areas only.

**Table 4:** The fully convolutional neural networks proposed in Lorenzo et al. (2019) have been trained using a number of datasets with different preprocessing and augmentations.

**Setup→**	**A, A'**	**B, B'**	**C, C'**	**D, D'**	**E, E'**	**F, F'**	**G, G'**	**H, H'**	**I, I'**	**J, J'**	**K, K'**	**L, L'**	**M, M'**	**N, N'**	**O, O'**
Feature centering	No	Yes	Yes	Yes	Yes	No	Yes	Yes	Yes	Yes	No	Yes	Yes	Yes	Yes
Vertical flip	No	No	Yes	No	Yes	No	No	Yes	No	Yes	No	No	Yes	No	Yes
Horizontal flip	No	No	No	Yes	Yes	No	No	No	Yes	Yes	No	No	No	Yes	Yes
Max. rotation (∡max)	0	0	0	0	0	45	45	45	45	45	90	90	90	90	90
Augmentation factor	1, 2	1, 2	2, 4	2, 4	4, 8	2, 4	2, 4	4, 8	4, 8	8, 16	2, 4	2, 4	4, 8	4, 8	8, 16

*In the prime versions, we applied elastic deformations. This table comes from our previous paper (Lorenzo et al., 2019)*.

**Figure 9:**
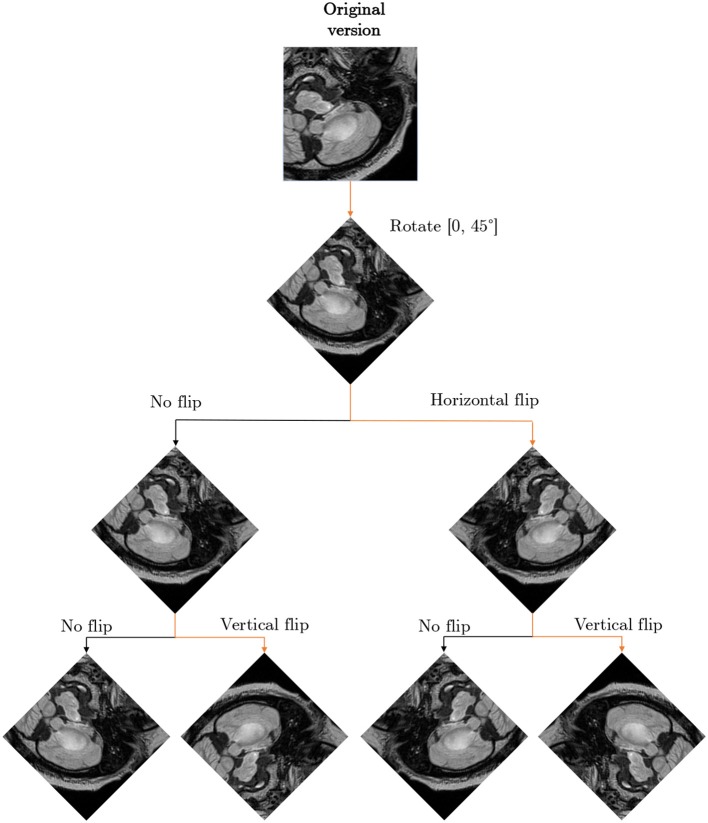
Exploiting various augmentations and coupling them into an augmentation tree allow us to generate multiple versions of an original patch (or image) which may be included in a training set. This figure is inspired by Lorenzo et al. (2019).

We executed multi-step augmentation by applying both affine and elastic deformations of tumor examples, and increased the cardinality of our training sets up to 16 ×. In Figure 9, we can observe how executing simple affine transformations leads to new synthetic image patches. Since various augmentation approaches may be utilized at different depths of this augmentation tree, the number of artificial examples can be significantly increased. The multi-fold cross-validation experiments showed that introducing rotated training examples was pivotal to boost the generalization abilities of underlying deep models. To verify the statistical importance of the results, we executed the Friedman's ranking tests which revealed that the horizontal flip with additional rotation is crucial to build well-generalizing deep learners in the patch-based segmentation scenario (Table 5).

**Table 5:** Five best-performing configurations of our fully convolutional neural network according to the Friedman's test (at *p* < 0.05) taking into account the results elaborated for the WHO II validation set (Lorenzo et al., 2019).

**Variant→**	**I**	**E**	**O**	**E'**	**J'**
Rank	4.75	5.50	6.00	7.25	7.75

**Figure 10:**
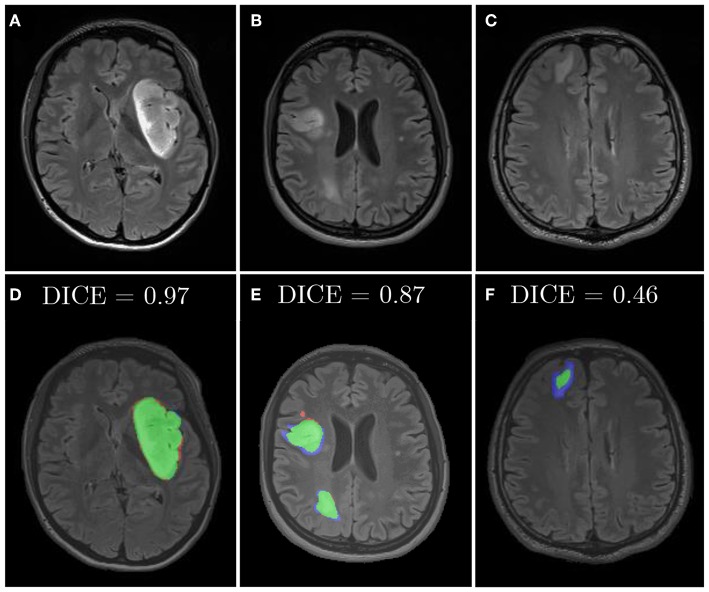
Examples from our clinical dataset segmented using our deep network trained in the DIR+Flip setting: **(A–C)** are original images, **(D–F)** are corresponding segmentations. Green color represents true positives, blue—false negatives, and red—false positives.

Similarly, we applied diffeomorphic image registration (DIR) coupled with a recommendation algorithm^6^ to select training image pairs for registration in the data augmentation process (Nalepa et al., 2019a). The proposed augmentation was compared with random horizontal flipping, and the experiments indicated that the combined approach leads to statistically significant (Wilcoxon test at *p* < 0.01) improvements in DICE (Table 6). In Figure 10, we have gathered example segmentations obtained using our DIR+Flip deep model, alongside the corresponding DICE values. Although the original network, trained over the original training set would correctly detect and segment large tumors (Figures 10A,B), it failed for relatively small lesions which were under-represented in the training set (Figure 10C). Similarly, synthesizing artificial training examples helped improving the performance of our models in the case of brain tumors located in the brain areas which have not been originally included in the dataset (by applying rotation and flipping).

**Table 6:** The results, both (a) average, and (b) median DICE over our clinical MRI data of low-grade glioma (WHO II) patients in the whole-tumor segmentation task, for different augmentation scenarios.

	**Augmentation**	**Training**	**Validation**	**Test**
(a)	Without	0.823	0.743	0.763
	Flip	0.836	0.790	0.785
	DIR	0.858	0.777	0.773
	DIR + Flip	**0.865**	**0.808**	**0.800**
(b)	Without	0.823	0.779	0.785
	Flip	0.838	0.808	0.797
	DIR	0.859	0.802	0.792
	DIR + Flip	**0.867**	**0.816**	**0.809**

*The results come from our paper (Nalepa et al., 2019a). The best results are boldfaced*.

## 5. Conclusion

In this paper, we reviewed the state-of-the-art data augmentation methods applied in the context of segmenting brain tumors from MRI. We carefully investigated all BraTS 2018 papers and analyzed data augmentation techniques utilized in these methods. Our investigation revealed that the affine transformations are still the most widely-used in practice, since they are trivial to implement and can elaborate anatomically-correct brain-tumor examples. There are, however, augmentation methods which combine various approaches, also including elastic transformations. A very interesting research direction encompasses algorithms which can generate artificial images (e.g., based on the tumoral growth models) that not necessarily follow real-life data distribution, but can be followed by other techniques to ensure correctness of such phantoms. The results showed that data augmentation was pivotal in the best-performing BraTS algorithms, and Isensee et al. (2018) experimentally proved that well-known and widely-used fully-convolutional neural networks can outperform other (perhaps much more deeper and complex) learners, if the training data is appropriately cleansed and curated. It clearly indicates the importance of introducing effective data augmentation methods for medical image data, which benefit from affine transformations (in 2D and 3D), pixel-wise modifications and elastic transform to deal with the problem of limited ground-truth data. In Table 7, we gather the advantages and disadvantages of all groups of brain-tumor data augmentation techniques analyzed in this review. Finally, these approaches can be easily applied in both single- and multi-modal scans, usually by synthesizing artificial examples separately for each image modality.

**Table 7:** The pros and cons of state-of-the-art brain-tumor data augmentation algorithms.

**Transformation of original data**	
**Advantages**	**Disadvantages**
***Affine transformations***	
• Easy to implement and understand	• Produce correlated images
• Operate in real-time due to low time complexity	• Easily generate anatomically incorrect examples (*)
• Applicable in training- and test-time	
• Deliver invariance with respect to the lesion position, scale, and rotation	
***Elastic transformations***	
• Can be applicable in training- and test-time	• Not trivial to implement
• Can introduce variations in shape	• Often have high time complexity
	• Easily generate anatomically incorrect examples (*)
***Pixel-wise transformations***	
• Easy to implement and understand	• Cannot introduce changes in shape
• Operate in real-time due to low time complexity	
• Applicable in training- and test-time	
• Can simulate different acquisition scenarios	
**Generation of artificial data**	
• Can synthesize realistic examples	• (Very) high time complexity
• (Potentially) applicable in test-time	• GANs applicable in training-time only
• Can introduce invariance with respect to affine transformations and appearance variations	• Can easily render multiple similar examples (mode collapse problem)

* *The real impact of incorporating unrealistic examples into training sets still needs investigation*.

**Figure 11:**
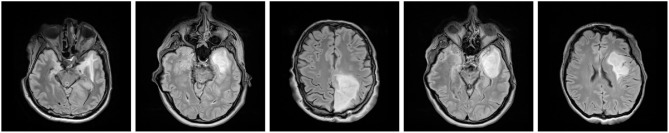
Anatomically incorrect brain images may still manifest valid tumor features—the impact of including such examples (which may be easily rendered by various data-generation augmentation techniques) into training sets for brain-tumor detection and segmentation tasks is yet to be revealed.

Although data augmentation became a pivotal part of virtually all deep learning-powered methods for segmenting brain lesions (due to the lack of very large, sufficiently heterogeneous and representative ground-truth sets, with BraTS being an exception), there are still promising and unexplored research pathways in the literature. We believe that hybridizing techniques from various algorithmic groups, introducing more data-driven augmentations, and applying them at training- and test-time can further boost the performance of large-capacity learners. Also, investigating the impact of including not necessarily anatomically correct brain-tumor scans into training sets remains an open issue (see the examples of anatomically incorrect brain images which still manifest valid tumor characteristics in Figure 11).

## Author Contributions

JN designed the study, performed the experiments, analyzed data, and wrote the manuscript. MM provided selected implementations and experimental results, and contributed to writing of some parts of the initial version of the manuscript. MK provided qualitative segmentation analysis and visualizations.

### Conflict of Interest

JN was employed by Future Processing, and MM was employed by Netguru. The remaining author declares that the research was conducted in the absence of any commercial or financial relationships that could be construed as a potential conflict of interest.

## Footnotes

^1^Test-time augmentation is also referred to as the *inference-time* and the *online* data augmentation in the literature.

^2^These variations can be however alleviated by appropriate data standardization.

^3^We tested the null hypothesis saying that applying the models trained exclusively over the BraTS or our WHO II datasets leads to the same-quality segmentation.

^4^Note that we do not count the algorithm proposed by Albiol et al. (2019), because the authors were not very specific about their augmentation strategies.

^5^For more detail on the validation and scoring procedures, see Bakas et al. (2018).

^6^We used a recommendation algorithm for selecting source-target image pairs that undergo registration. Such pairs should contain the training images which capture lesions positioned in the same or close part of the brain, as the totally different images can easily render unrealistic brain-tumor examples. A potential drawback of this recommendation technique is its time complexity which amounts to $O\left(||T|{|}^{2}\right)$, where ||T|| is the cardinality of the original training set.

## References

[B1] Agarwal M. Mahajan R. (2017). Medical images contrast enhancement using quad weighted histogram equalization with adaptive gama correction and homomorphic filtering. Proc. Comput. Sci. 115, 509–517. 10.1016/j.procs.2017.09.107

[B2] Albiol A. Albiol A. Albiol F. (2019). “Extending 2D deep learning architectures to 3D image segmentation problems,” in Brainlesion: Glioma, Multiple Sclerosis, Stroke and Traumatic Brain Injuries, eds Crimi S. Bakas A. Kuijf H. Keyvan F. Reyes M. van Walsum T. (Cham: Springer International Publishing), 73–82.

[B3] Alex V. Mohammed Safwan K. P. Chennamsetty S. S. Krishnamurthi G. (2017). “Generative adversarial networks for brain lesion detection,” in Medical Imaging 2017: Image Processing, eds Styner M. A. Angelini E. D. (SPIE), 113–121. 10.1117/12.2254487

[B4] Amit G. Ben-Ari R. Hadad O. Monovich E. Granot N. Hashoul S. (2017). “Classification of breast MRI lesions using small-size training sets: comparison of deep learning approaches,” in Medical Imaging 2017: Computer-Aided Diagnosis, eds Armato S. G. III Petrick N. A. (SPIE), 374–379. 10.1117/12.2249981

[B5] Angulakshmi M. Lakshmi Priya G. (2017). Automated brain tumour segmentation techniques—a review. Int. J. Imaging Syst. Technol. 27, 66–77. 10.1002/ima.22211

[B6] Asif U. Bennamoun M. Sohel F. A. (2018). A multi-modal, discriminative and spatially invariant CNN for RGB-D object labeling. IEEE Trans. Patt. Anal. Mach. Intell. 40, 2051–2065. 10.1109/TPAMI.2017.2747134 28866483

[B7] Bakas S. Akbari H. Sotiras A. Bilello M. Rozycki M. Kirby J. S. (2017a). Segmentation labels and radiomic features for the pre-operative scans of the TCGA-GBM collection. Cancer Imaging Arch. Available online at: https://wiki.cancerimagingarchive.net/display/DOI/Segmentation+Labels+and+Radiomic+Features+for+the+Pre-operative+Scans+of+the+TCGA-GBM+collection

[B8] Bakas S. Akbari H. Sotiras A. Bilello M. Rozycki M. Kirby J. S. (2017b). Segmentation labels and radiomic features for the pre-operative scans of the TCGA-LGG collection. Cancer Imaging Arch. Available online at: https://wiki.cancerimagingarchive.net/display/DOI/Segmentation+Labels+and+Radiomic+Features+for+the+Pre-operative+Scans+of+the+TCGA-GBM+collection

[B9] Bakas S. Akbari H. Sotiras A. Bilello M. Rozycki M. Kirby J. S.. (2017c). Advancing the cancer genome atlas glioma MRI collections with expert segmentation labels and radiomic features. Sci. Data 4, 1–13. 10.1038/sdata.2017.117 28872634 PMC5685212

[B10] Bakas S. Reyes M. Jakab A. Bauer S. Rempfler M. Crimi A. (2018). Identifying the best machine learning algorithms for brain tumor segmentation, progression assessment, and overall survival prediction in the BRATS challenge. CoRR abs/1811.02629.

[B11] Bengio Y. Courville A. Vincent P. (2013). Representation learning: a review and new perspectives. IEEE TPAMI 35, 1798–1828. 10.1109/TPAMI.2013.50 23787338

[B12] Benson E. Pound M. P. French A. P. Jackson A. S. Pridmore T. P. (2018). “Deep hourglass for brain tumor segmentation,” in Brainlesion: Glioma, Multiple Sclerosis, Stroke and Traumatic Brain Injuries - 4th International Workshop, BrainLes 2018, Held in Conjunction with MICCAI 2018, Granada, Spain, September 16, 2018, Revised Selected Papers, Part II, vol. 11384 of Lecture Notes in Computer Science (Cham), 419–428.

[B13] Carver E. Liu C. Zong W. Dai Z. Snyder J. M. Lee J. (2018). “Automatic brain tumor segmentation and overall survival prediction using machine learning algorithms,” in Brainlesion: Glioma, Multiple Sclerosis, Stroke and Traumatic Brain Injuries - 4th International Workshop, BrainLes 2018, Held in Conjunction with MICCAI 2018, Granada, Spain, September 16, 2018, Revised Selected Papers, Part II, vol. 11384 of Lecture Notes in Computer Science (Cham), 406–418.

[B14] Castro E. Cardoso J. S. Pereira J. C. (2018). “Elastic deformations for data augmentation in breast cancer mass detection,” in 2018 IEEE EMBS International Conference on Biomedical Health Informatics (BHI), 230–234. 10.1109/BHI.2018.8333411

[B15] Chaitanya K. Karani N. Baumgartner C. F. Becker A. Donati O. Konukoglu E. (2019). “Semi-supervised and task-driven data augmentation,” in Information Processing in Medical Imaging, eds Chung A. C. S. Gee J. C. Yushkevich P. A. Bao S. (Cham: Springer International Publishing), 29–41.

[B16] Chandra S. Vakalopoulou M. Fidon L. Battistella E. Estienne T. Sun R. (2018). “Context aware 3D CNNs for brain tumor segmentation,” in Brainlesion: Glioma, Multiple Sclerosis, Stroke and Traumatic Brain Injuries - 4th International Workshop, BrainLes 2018, Held in Conjunction with MICCAI 2018, Granada, Spain, September 16, 2018, Revised Selected Papers, Part II, vol. 11384 of Lecture Notes in Computer Science (Cham), 299–310.

[B17] Crimi A. Bakas S. Kuijf H. J. Keyvan F. Reyes M. van Walsum T. (eds). (2019). Brainlesion: Glioma, Multiple Sclerosis, Stroke and Traumatic Brain Injuries - 4th International Workshop, BrainLes 2018, Held in Conjunction with MICCAI 2018, Granada, Spain, September 16, 2018, Revised Selected Papers, Part II, volume 11384 of Lecture Notes in Computer Science (Cham).

[B18] Dai L. Li T. Shu H. Zhong L. Shen H. Zhu H. (2018). “Automatic brain tumor segmentation with domain adaptation,” in Brainlesion: Glioma, Multiple Sclerosis, Stroke and Traumatic Brain Injuries - 4th International Workshop, BrainLes 2018, Held in Conjunction with MICCAI 2018, Granada, Spain, September 16, 2018, Revised Selected Papers, Part II, vol. 11384 of Lecture Notes in Computer Science (Cham), 380–392.

[B19] Dvornik N. Mairal J. Schmid C. (2018). On the importance of visual context for data augmentation in scene understanding. CoRR abs/1809.02492. 10.1109/TPAMI.2019.2961896 31880540

[B20] Eaton-Rosen Z. Bragman F. Ourselin S. Cardoso M. J. (2019). Improving data augmentation for medical image segmentation. OpenReview.

[B21] Feng X. Tustison N. J. Meyer C. H. (2018). “Brain tumor segmentation using an ensemble of 3d u-nets and overall survival prediction using radiomic features,” in Brainlesion: Glioma, Multiple Sclerosis, Stroke and Traumatic Brain Injuries - 4th International Workshop, BrainLes 2018, Held in Conjunction with MICCAI 2018, Granada, Spain, September 16, 2018, Revised Selected Papers, Part II, vol. 11384 of Lecture Notes in Computer Science (Cham), 279–288.

[B22] Frid-Adar M. Diamant I. Klang E. Amitai M. Goldberger J. Greenspan H. (2018). Gan-based synthetic medical image augmentation for increased cnn performance in liver lesion classification. Neurocomputing 321, 321–331. 10.1016/j.neucom.2018.09.013

[B23] Fyllingen E. H. Stensjøen A. L. Berntsen E. M. Solheim O. Reinertsen I. (2016). Glioblastoma segmentation: comparison of three different software packages. PLoS ONE 11:e0164891. 10.1371/journal.pone.0164891 27780224 PMC5079567

[B24] Galdran A. Alvarez-Gila A. Meyer M. I. Saratxaga C. L. Araujo T. Garrote E. (2017). Data-driven color augmentation techniques for deep skin image analysis. CoRR abs/1703.03702.

[B25] Gholami A. Subramanian S. Shenoy V. Himthani N. Yue X. Zhao S. (2018). “A novel domain adaptation framework for medical image segmentation,” in Brainlesion: Glioma, Multiple Sclerosis, Stroke and Traumatic Brain Injuries - 4th International Workshop, BrainLes 2018, Held in Conjunction with MICCAI 2018, Granada, Spain, September 16, 2018, Revised Selected Papers, Part II, vol. 11384 of Lecture Notes in Computer Science (Cham), 289–298.

[B26] Gibson E. Li W. Sudre C. Fidon L. Shakir D. I. Wang G.. (2018). NiftyNet: a deep-learning platform for medical imaging. Comput. Methods Prog. Biomed. 158, 113–122. 10.1016/j.cmpb.2018.01.025 29544777 PMC5869052

[B27] Goodfellow I. Pouget-Abadie J. Mirza M. Xu B. Warde-Farley D. Ozair S. (2014). “Generative adversarial nets,” in Advances in Neural Information Processing Systems 27, eds Ghahramani Z. Welling M. Cortes C. Lawrence N. D. Weinberger K. Q. (Curran Associates, Inc), 2672–2680. Available online at: http://papers.nips.cc/paper/5423-generative-adversarial-nets.pdf

[B28] Gu S. Meng X. Sciurba F. C. Ma H. Leader J. Kaminski N.. (2014). Bidirectional elastic image registration using b-spline affine transformation. Comput. Med. Imaging Graph. 38, 306–314. 10.1016/j.compmedimag.2014.01.002 24530210 PMC4019704

[B29] Han C. Murao K. Satoh S. Nakayama H. (2019). Learning more with less: gan-based medical image augmentation. CoRR abs/1904.00838. 10.1145/3357384.3357890

[B30] Hollingworth W. Medina L. S. Lenkinski R. E. Shibata D. K. Bernal B. Zurakowski D.. (2006). Interrater reliability in assessing quality of diagnostic accuracy studies using the quadas tool: a preliminary assessment. Acad. Radiol. 13, 803–810. 10.1016/j.acra.2006.03.008 16777553

[B31] Huang Z. Cohen F. S. (1996). Affine-invariant b-spline moments for curve matching. IEEE Trans. Image Process. 5, 1473–1480. 10.1109/83.536895 18290064

[B32] Hussain Z. Gimenez F. Yi D. Rubin D. (2017). “Differential data augmentation techniques for medical imaging classification tasks,” in AMIA 2017, American Medical Informatics Association Annual Symposium (Washington, DC). PMC5977656 29854165

[B33] Isensee F. Kickingereder P. Wick W. Bendszus M. Maier-Hein K. H. (2018). “No new-net,” in Brainlesion: Glioma, Multiple Sclerosis, Stroke and Traumatic Brain Injuries - 4th International Workshop, BrainLes 2018, Held in Conjunction with MICCAI 2018, Granada, Spain, September 16, 2018, Revised Selected Papers, Part II, vol. 11384 of Lecture Notes in Computer Science (Cham), 234–244.

[B34] Isin A. Direkoglu C. Sah M. (2016). Review of mri-based brain tumor image segmentation using deep learning methods. Proc. Comput. Sci. 102, 317–324. 10.1016/j.procs.2016.09.407

[B35] Kao P. Ngo T. Zhang A. Chen J. W. Manjunath B. S. (2018). “Brain tumor segmentation and tractographic feature extraction from structural MR images for overall survival prediction,” in Brainlesion: Glioma, Multiple Sclerosis, Stroke and Traumatic Brain Injuries - 4th International Workshop, BrainLes 2018, Held in Conjunction with MICCAI 2018, Granada, Spain, September 16, 2018, Revised Selected Papers, Part II, vol. 11384 of Lecture Notes in Computer Science (Cham), 128–141.

[B36] Kermi A. Mahmoudi I. Khadir M. T. (2018). “Deep convolutional neural networks using u-net for automatic brain tumor segmentation in multimodal MRI volumes,” in Brainlesion: Glioma, Multiple Sclerosis, Stroke and Traumatic Brain Injuries - 4th International Workshop, BrainLes 2018, Held in Conjunction with MICCAI 2018, Granada, Spain, September 16, 2018, Revised Selected Papers, Part II, vol. 11384 of Lecture Notes in Computer Science (Cham), 37–48.

[B37] Krizhevsky A. Sutskever I. Hinton G. E. (2017). Imagenet classification with deep convolutional neural networks. Commun. ACM 60, 84–90. 10.1145/3065386

[B38] Lachinov D. Vasiliev E. Turlapov V. (2018). “Glioma segmentation with cascaded unet,” in Brainlesion: Glioma, Multiple Sclerosis, Stroke and Traumatic Brain Injuries - 4th International Workshop, BrainLes 2018, Held in Conjunction with MICCAI 2018, Granada, Spain, September 16, 2018, Revised Selected Papers, Part II, vol. 11384 of Lecture Notes in Computer Science (Cham), 189–198.

[B39] LeCun Y. Bengio Y. Hinton G. (2016). Deep learning. Nature 521, 436–555. 10.1038/nature14539 26017442

[B40] Litjens G. Kooi T. Bejnordi B. E. Setio A. A. A. Ciompi F. Ghafoorian M.. (2017). A survey on deep learning in medical image analysis. Med. Image Anal. 42, 60–88. 10.1016/j.media.2017.07.005 28778026

[B41] Liu Y. Stojadinovic S. Hrycushko B. Wardak Z. Lau S. Lu W.. (2017). A deep convolutional neural network-based automatic delineation strategy for multiple brain metastases stereotactic radiosurgery. PLoS ONE 12:e0185844. 10.1371/journal.pone.0185844 28985229 PMC5630188

[B42] Ma J. Yang X. (2018). “Automatic brain tumor segmentation by exploring the multi-modality complementary information and cascaded 3d lightweight cNNs,” in Brainlesion: Glioma, Multiple Sclerosis, Stroke and Traumatic Brain Injuries - 4th International Workshop, BrainLes 2018, Held in Conjunction with MICCAI 2018, Granada, Spain, September 16, 2018, Revised Selected Papers, Part II, vol. 11384 of Lecture Notes in Computer Science (Cham), 25–36.

[B43] Marcinkiewicz M. Nalepa J. Lorenzo P. R. Dudzik W. Mrukwa G. (2018). “Segmenting brain tumors from MRI using cascaded multi-modal U-Nets,” in Brainlesion: Glioma, Multiple Sclerosis, Stroke and Traumatic Brain Injuries - 4th International Workshop, BrainLes 2018, Held in Conjunction with MICCAI 2018, Granada, Spain, September 16, 2018, Revised Selected Papers, Part II, vol. 11384 of Lecture Notes in Computer Science (Cham), 13–24.

[B44] McKinley R. Meier R. Wiest R. (2018). “Ensembles of densely-connected cnns with label-uncertainty for brain tumor segmentation,” in Brainlesion: Glioma, Multiple Sclerosis, Stroke and Traumatic Brain Injuries - 4th International Workshop, BrainLes 2018, Held in Conjunction with MICCAI 2018, Granada, Spain, September 16, 2018, Revised Selected Papers, Part II, vol. 11384 of Lecture Notes in Computer Science (Cham), 456–465.

[B45] Mehta R. Arbel T. (2018). “3D U-Net for brain tumour segmentation,” in Brainlesion: Glioma, Multiple Sclerosis, Stroke and Traumatic Brain Injuries - 4th International Workshop, BrainLes 2018, Held in Conjunction with MICCAI 2018, Granada, Spain, September 16, 2018, Revised Selected Papers, Part II, vol. 11384 of Lecture Notes in Computer Science (Cham), 254–266.

[B46] Menze B. H. Jakab A. Bauer S. Kalpathy-Cramer J. Farahani K. Kirby J.. (2015). The multimodal brain tumor image segmentation benchmark (BraTS). IEEE TMI 34, 1993–2024. 10.1109/TMI.2014.2377694 25494501 PMC4833122

[B47] Mok T. C. W. Chung A. C. S. (2018). Learning data augmentation for brain tumor segmentation with coarse-to-fine generative adversarial networks. CoRR abs/1805.11291:1–10.

[B48] Myronenko A. (2018). “3D MRI brain tumor segmentation using autoencoder regularization,” in Brainlesion: Glioma, Multiple Sclerosis, Stroke and Traumatic Brain Injuries - 4th International Workshop, BrainLes 2018, Held in Conjunction with MICCAI 2018, Granada, Spain, September 16, 2018, Revised Selected Papers, Part II, vol. 11384 of Lecture Notes in Computer Science (Cham), 311–320.

[B49] Nalepa J. Lorenzo P. R. Marcinkiewicz M. Bobek-Billewicz B. Wawrzyniak P. Walczak M. (2019). Fully-automated deep learning-powered system for DCE-MRI analysis of brain tumors. CoRR abs/1907.08303. 10.1016/j.artmed.2019.101769 31980106

[B50] Nalepa J. Mrukwa G. Piechaczek S. Lorenzo P. R. Marcinkiewicz M. Bobek-Billewicz B. (2019a). “Data augmentation via image registration,” in 2019 IEEE International Conference on Image Processing (ICIP), 4250–4254.

[B51] Nalepa J. Myller M. Kawulok M. (2019b). Training- and test-time data augmentation for hyperspectral image segmentation. IEEE Geosci. Remote Sens. Lett. 1–5. 10.1109/LGRS.2019.2921011

[B52] Nguyen K. P. Fatt C. C. Treacher A. Mellema C. Trivedi M. H. Montillo A. (2019). Anatomically-informed data augmentation for functional MRI with applications to deep learning. CoRR abs/1910.08112. 10.1117/12.2548630 PMC7990266 33767520

[B53] Nuechterlein N. Mehta S. (2018). “3D-ESPNet with pyramidal refinement for volumetric brain tumor image segmentation,” in Brainlesion: Glioma, Multiple Sclerosis, Stroke and Traumatic Brain Injuries - 4th International Workshop, BrainLes 2018, Held in Conjunction with MICCAI 2018, Granada, Spain, September 16, 2018, Revised Selected Papers, Part II, vol. 11384 of Lecture Notes in Computer Science (Cham), 245–253.

[B54] Oksuz I. Ruijsink B. Puyol-Antón E. Clough J. R. Cruz G. Bustin A.. (2019). Automatic CNN-based detection of cardiac MR motion artefacts using k-space data augmentation and curriculum learning. Med. Image Anal. 55, 136–147. 10.1016/j.media.2019.04.009 31055126 PMC6688894

[B55] Park S.-C. Cha J. H. Lee S. Jang W. Lee C. S. Lee J. K. (2019). Deep learning-based deep brain stimulation targeting and clinical applications. Front. Neurosci. 13:1128. 10.3389/fnins.2019.01128 31708729 PMC6821714

[B56] Pereira S. Pinto A. Alves V. Silva C. A. (2016). Brain tumor segmentation using convolutional neural nets in MRI images. IEEE TMI 35, 1240–1251. 10.1109/TMI.2016.2538465 26960222

[B57] Puybareau É. Tochon G. Chazalon J. Fabrizio J. (2018). “Segmentation of gliomas and prediction of patient overall survival: a simple and fast procedure,” in Brainlesion: Glioma, Multiple Sclerosis, Stroke and Traumatic Brain Injuries - 4th International Workshop, BrainLes 2018, Held in Conjunction with MICCAI 2018, Granada, Spain, September 16, 2018, Revised Selected Papers, Part II, vol. 11384 of Lecture Notes in Computer Science (Cham), 199–209.

[B58] Rezaei M. Harmuth K. Gierke W. Kellermeier T. Fischer M. Yang H. (2017). Conditional adversarial network for semantic segmentation of brain tumor. CoRR abs/1708.05227:1–10.

[B59] Rezaei M. Yang H. Meinel C. (2018). “voxel-gan: adversarial framework for learning imbalanced brain tumor segmentation,” in Brainlesion: Glioma, Multiple Sclerosis, Stroke and Traumatic Brain Injuries - 4th International Workshop, BrainLes 2018, Held in Conjunction with MICCAI 2018, Granada, Spain, September 16, 2018, Revised Selected Papers, Part II, vol. 11384 of Lecture Notes in Computer Science (Cham), 321–333.

[B60] Ribalta Lorenzo P. Nalepa J. Bobek-Billewicz B. Wawrzyniak P. Mrukwa G. Kawulok M.. (2019). Segmenting brain tumors from flair MRI using fully convolutional neural networks. Comput. Methods Prog. Biomed. 176, 135–148. 10.1016/j.cmpb.2019.05.006 31200901

[B61] Rozsa A. Günther M. Boult T. E. (2016). Towards robust deep neural networks with BANG. CoRR abs/1612.00138.

[B62] Sahnoun M. Kallel F. Dammak M. Mhiri C. Ben Mahfoudh K. Ben Hamida A. (2018). “A comparative study of MRI contrast enhancement techniques based on Traditional Gamma Correction and Adaptive Gamma Correction: Case of multiple sclerosis pathology,” in 2018 4th International Conference on Advanced Technologies for Signal and Image Processing (ATSIP), 1–7.

[B63] Sauwen N. Acou M. Sima D. M. Veraart J. Maes F. Himmelreich U.. (2017). Semi-automated brain tumor segmentation on multi-parametric MRI using regularized non-negative matrix factorization. BMC Med. Imaging 17:29. 10.1186/s12880-017-0198-4 28472943 PMC5418702

[B64] Shin H.-C. Tenenholtz N. A. Rogers J. K. Schwarz C. G. Senjem M. L. Gunter J. L. (2018). “Medical image synthesis for data augmentation and anonymization using generative adversarial networks,” in Simulation and Synthesis in Medical Imaging, eds Gooya A. Goksel O. Oguz I. Burgos N. (Cham: Springer), 1–11.

[B65] Shorten C. Khoshgoftaar T. M. (2019). A survey on image data augmentation for deep learning. J. Big Data 6:60 10.1186/s40537-019-0197-0 PMC8287113 34306963

[B66] Sun L. Zhang S. Luo L. (2018). “Tumor segmentation and survival prediction in glioma with deep learning,” in Brainlesion: Glioma, Multiple Sclerosis, Stroke and Traumatic Brain Injuries - 4th International Workshop, BrainLes 2018, Held in Conjunction with MICCAI 2018, Granada, Spain, September 16, 2018, Revised Selected Papers, Part II, vol. 11384 of Lecture Notes in Computer Science (Cham), 83–93.

[B67] Tustison N. J. Avants B. B. (2013). Explicit B-spline regularization in diffeomorphic image registration. Front. Neuroinformatics 7:39. 10.3389/fninf.2013.00039 24409140 PMC3870320

[B68] Tustison N. J. Avants B. B. Gee J. C. (2009). Directly manipulated free-form deformation image registration. IEEE TIP 18, 624–635. 10.1109/TIP.2008.2010072 19171516

[B69] Tward D. Miller M. (2017). “Unbiased diffeomorphic mapping of longitudinal data with simultaneous subject specific template estimation,” in Graphs in Biomedical Image Analysis, Computational Anatomy and Imaging Genetics, eds Cardoso M. J. Arbel T. Ferrante E. Pennec X. Dalca A. V. Parisot S. Joshi S. Batmanghelich N. K. Sotiras A. Nielsen M. Sabuncu M. R. Fletcher T. Shen L. Durrleman S. Sommer S. (Cham: Springer International Publishing), 125–136.

[B70] Visser M. Müller D. M. J. van Duijn R. J. M. Smits M. Verburg N. Hendriks E. J.. (2019). Inter-rater agreement in glioma segmentations on longitudinal MRI. NeuroImage 22:101727. 10.1016/j.nicl.2019.101727 30825711 PMC6396436

[B71] Wang G. Li W. Aertsen M. Deprest J. Ourselin S. Vercauteren T. (2019). Aleatoric uncertainty estimation with test-time augmentation for medical image segmentation with convolutional neural networks. Neurocomputing 338, 34–45. 10.1016/j.neucom.2019.01.103 PMC6783308 31595105

[B72] Wang G. Li W. Ourselin S. Vercauteren T. (2018). “Automatic brain tumor segmentation using convolutional neural networks with test-time augmentation,” in Brainlesion: Glioma, Multiple Sclerosis, Stroke and Traumatic Brain Injuries - 4th International Workshop, BrainLes 2018, Held in Conjunction with MICCAI 2018, Granada, Spain, September 16, 2018, Revised Selected Papers, Part II, vol. 11384 of Lecture Notes in Computer Science (Cham), 61–72.

[B73] Wang K. Gou C. Duan Y. Lin Y. Zheng X. Wang F. (2017). Generative adversarial networks: introduction and outlook. IEEE/CAA J. Automat. Sin. 4, 588–598. 10.1109/JAS.2017.7510583

[B74] Wei W. Liu L. Truex S. Yu L. Gursoy M. E. (2018). Adversarial examples in deep learning: characterization and divergence. CoRR abs/1807.00051.

[B75] Wong S. C. Gatt A. Stamatescu V. McDonnell M. D. (2016). “Understanding data augmentation for classification: when to warp?” in 2016 International Conference on Digital Image Computing: Techniques and Applications (DICTA), 1–6. 10.1109/DICTA.2016.7797091

[B76] Yu B. Zhou L. Wang L. Fripp J. Bourgeat P. (2018). “3D cGAN based cross-modality MR image synthesis for brain tumor segmentation,” in 2018 IEEE 15th International Symposium on Biomedical Imaging (ISBI 2018), 626–630. 10.1109/ISBI.2018.8363653

[B77] Zhang H. Cissé M. Dauphin Y. N. Lopez-Paz D. (2017). mixup: beyond empirical risk minimization. CoRR abs/1710.09412.

[B78] Zhao J. Meng Z. Wei L. Sun C. Zou Q. Su R. (2019). Supervised brain tumor segmentation based on gradient and context-sensitive features. Front. Neurosci. 13:144. 10.3389/fnins.2019.00144 30930729 PMC6427904

[B79] Zhu J. Park T. Isola P. Efros A. A. (2017). Unpaired image-to-image translation using cycle-consistent adversarial networks. CoRR abs/1703.10593. 10.1109/ICCV.2017.244

